# Exploring the Enteric Virome of Cats with Acute Gastroenteritis

**DOI:** 10.3390/vetsci10050362

**Published:** 2023-05-18

**Authors:** Federica Di Profio, Vittorio Sarchese, Paola Fruci, Giovanni Aste, Vito Martella, Andrea Palombieri, Barbara Di Martino

**Affiliations:** 1 Department of Veterinary Medicine, Università degli Studi di Teramo, 64100 Teramo, Italy; fdiprofio@unite.it (F.D.P.); vsarchese@unite.it (V.S.); pfruci@unite.it (P.F.); gaste@unite.it (G.A.); bdimartino@unite.it (B.D.M.); 2 Department of Veterinary Medicine, Università Aldo Moro di Bari, 70010 Valenzano, Italy; vito.martella@uniba.it

**Keywords:** gastroenteritis, cats, viruses, PCRs and RT-PCRs, ONT sequencing

## Abstract

**Simple Summary:**

Acute gastroenteritis (AGE) is a very common illness and a significant cause of morbidity and mortality in cats less than one year of age. Along with known viruses historically recognized as primary agents of AGE, in the last years deep sequencing technologies have identified a variety of novel viruses. In this study, we explored the feline enteric virome from cats with or without diarrhoea, unveiling a wide diversity of viruses.

**Abstract:**

Viruses are a major cause of acute gastroenteritis (AGE) in cats, chiefly in younger animals. Enteric specimens collected from 29 cats with acute enteritis and 33 non-diarrhoeic cats were screened in PCRs and reverse transcription (RT) PCR for a large panel of enteric viruses, including also orphan viruses of recent identification. At least one viral species, including feline panleukopenia virus (FPV), feline enteric coronavirus (FCoV), feline chaphamaparvovirus, calicivirus (vesivirus and novovirus), feline kobuvirus, feline sakobuvirus A and Lyon IARC polyomaviruses, was detected in 66.1% of the samples.. Co-infections were mainly accounted for by FPV and FCoV and were detected in 24.2% of the samples. The virome composition was further assessed in eight diarrhoeic samples, through the construction of sequencing libraries using a sequence-independent single-primer amplification (SISPA) protocol. The libraries were sequenced on Oxford Nanopore Technologies sequencing platform. A total of 41 contigs (>100 nt) were detected from seven viral families infecting mammals, included *Parvoviridae*, *Caliciviridae*, *Picornaviridae*, *Polyomaviridae*, *Anelloviridae*, *Papillomaviridae* and *Paramyxoviridae*, revealing a broad variety in the composition of the feline enteric virome.

## 1. Introduction

Acute gastroenteritis (AGE) in cats remains a leading cause of morbidity and mortality, chiefly in animals less than one year of age living in densely housed environments, such as kennels and animal shelters [1]. AGE can be caused by a broad spectrum of enteric pathogens, but in kittens, a large proportion of cases are due to enteric viruses, with feline panleukopenia virus (FPV) recognised as the main cause worldwide [2,3]. In recent years, advances in molecular diagnostics, based on the use of pan-viral PCRs with consensus-degenerate primers, have proven useful for discovery, including of many novel parvoviruses, caliciviruses and picornaviruses [4,5,6]. Furthermore, high-throughput sequencing-based investigations have provided significant clues to understand the variety of viral communities inhabiting the feline enteric environment. Unbiased analysis of nucleic acid from diarrhoeic samples of cats has enabled identification and characterization of previously unknown viruses such as feline sakobuvirus A (SaKoV-A), feline chaphamaparvovirus (FeChPV) and Lyon IARC polyomaviruses (LIPyVs) [7,8,9].

Observational studies and animal experiments to confirm their enteropathogenic role have been carried out for only a few viruses, such as feline astroviruses (FAstVs) [10] and feline noroviruses (FeNoVs) [11,12]. Alternatively, many of the novel discovered enteric viruses can still be considered as “orphan”, since the association between infection and diarrhoea remains uncertain. Additionally, the shift in microbiological laboratories to a syndromic diagnostic based on advanced multiplex assays for a large panel of pathogens is unveiling the frequency of mixed infections, posing challenges for the interpretation of results [13,14,15,16,17,18,19].

Herein, we report the results of a molecular survey aimed to investigate the presence of selected known and emerging/novel enteric viruses in the stool samples of cats by implementing the diagnostic algorithm of all cases of AGE admitted to the veterinary hospital of the Department of Veterinary Medicine, the University of Teramo (Italy). In addition, to further characterize the viral community associated with feline enteric disease, eight diarrhoeic specimens were assessed by combining a sequence-independent single-primer amplification (SISPA) approach with Oxford Nanopore Technologies (ONT) sequencing platform.

## 2. Materials and Methods

### 2.1. Sampling

A total of 62 enteric samples were collected, over a 7 month period (October 2020–May 2021), from young (2–12 months) and adult (older than 1 year) stray cats admitted to the veterinary hospital of the Department of Veterinary Medicine, University of Teramo (Teramo, Italy). Clinical signs of diarrhoea consistent with acute enteritis were observed in 29 animals whereas 33 cats, enrolled as control group, were hospitalized for surgical or other medical pathologies. As injured or sick free-ranging animals were also hospitalized in the framework of a regional project for emergency treatments and care, the vaccination status of all cats was unknown. In compliance with biosecurity measures, animal flow requires that all these cats, considered potentially infected, are kept temporarily in the quarantine area, whilst animals showing signs of suspected infectious disease are housed in the isolation area. Confirmed infected cats are moved to the infectious disease room. In each area, animals are retained in separate cages but under conditions in which they may have indirect contact with each other.

### 2.2. Virological Investigation

Viral nucleic acids were extracted from each faecal specimen using the TRIzol LS (Invitrogen, Ltd., Paisley, UK), following the manufacturer’s instructions. All the samples were screened, using specific or broadly reactive consensus PCR and reverse transcription (RT)-PCR assays at the family and/or genus level, for a selected panel of feline viral pathogens, including DNA viruses as FPV [20], feline bocaparvoviruses (FBoVs) [4], feline bufavirus (FBuV) [21], FeChPV [8], PyVs [22] and RNA viruses as feline enteric coronavirus (FCoV) [23,24], feline caliciviruses (FCV) [25], NoVs [26], AstVs [27], feline kobuvirus (FeKoV) [28] and SaKoV [29]. All primers used in this study are listed in Table 1. RT-PCR and PCR assays were performed using SuperScript ™ One-Step RT-PCR (Invitrogen, Ltd., Paisley, UK) and GoTaq^®^ Green Master Mix 1 (Promega Italia S.r.l, Milan, Italy), respectively. The amplification products were analysed using 1.5% agarose gel electrophoresis and visualized by UV after ethidium bromide staining. All of the amplicons yielding bands of expected size were excised from gel, purified with a QIAquick gel extraction kit (Qiagen GmbH, Hilden, Germany) and subjected to direct sequencing from both directions using BigDye Terminator Cycle chemistry and 3730 DNA Analyzer (Applied Biosystems, Foster, CA, USA). Basic Local Alignment Search Tool (BLAST; http://www.ncbi.nlm.nih.gov, accessed on 15 January 2023) and FASTA (http://www.ebi.ac.uk/fasta33, accessed on 15 January 2023) with default values were used to find homologous hits.

### 2.3. Statistical Analysis

The association of the clinical status with the presence of viral pathogens was assessed using Fisher’s exact test with GraphPad Prism Software (https://www.graphpad.com/scientific-software/prism/, accessed on 15 February 2023). The significance level of the test was set at *p* < 0.05.

### 2.4. SISPA, ONT Library Preparation and Sequencing

A SISPA-based enrichment and nanopore sequencing were applied to further investigate the virome composition of eight diarrhoeic specimens. Briefly, extracted DNA (20 µL) was mixed with 25 µL of NEBuffer™ 2 (New England Biolabs, Hitchin, UK), 1 µL of dNTPs at 10 mM (Invitrogen, Ltd., Paisley, UK) and 2 µL of primer FR26RV-N(GCCGGAGCTCTGCAGATATCNNNNNN) at 10 µM. The reaction was incubated at 94 °C for 2 min and cooled on ice for 2 min. Subsequently, 2.5 units (0.5 µL) of Klenow Fragment (3′→5′ exo-) (New England BioLabs, Hitchin, UK) were added and the reaction was incubated at 37 °C for 1 h. This cycle was repeated once and then followed by an enzyme inactivation step at 75 °C for 10 min. DNase-treated RNA was reverse transcribed to single-strand cDNA by Super-Script IV Reverse Transcriptase (Invitrogen Ltd., Milan, Italy) using primers FR26RV-N (GCCGGAGCTCTGCAGATATC-N6) [30] and FR40RV-T (GCCGGAGCTCTGCAGATATCTTTTTTTTTTTTTTTTTTTT) [31]. Second-strand cDNA synthesis was performed using Klenow Fragment (3′→5′ exo-) (New England BioLabs, Hitchin, UK). Of each reaction mix, 5 µL was used as a template in a subsequent PCR. The 50 µL reaction mix consisted of 10 µL of 5X Q5 Reaction Buffer (New England BioLabs, Hitchin, UK), 1 µL of dNTPs, 1 µL of primer FR20RV (GCCGGAGCTCTGCAGATATC) [30] and 1 µL of Q5 High-Fidelity DNA Polymerase at 0.04 U/µL (New England BioLabs, Hitchin, UK). After 2 min of activation at 94 °C, 40 cycles of amplification (94 °C for 30 s, 48 °C for 30 s, 68 °C for 8 min) and a final extension at 68 °C for 8 min were performed. PCR products were cleaned up with AMPure XP beads (Beckman Coulter, Milan, Italy) and quantified with a Qubit 4.0 instrument using a dsDNA HS Assay Kit (Thermo Fisher Scientific, Waltham, MA, USA). Libraries were prepared following manufacturer instructions by using the PCR Barcoding Expansion 1–12 (EXP-PBC001) kit with the Ligation Sequencing Kit (SQK-LSK-109), allowing multiple samples to be run on one R9 version flow cell (FLO-MIN106D, ONT). A nanopore library was run on MinION Mk1C device (ONT, Oxford, UK) for nine hours. On-site and real-time basecalling and demultiplexing during the sequencing run were carried out with ONT cloud GUPPY (v3.2.8) basecaller provided in the MinKNOW software package (v3.1.5).

The online tool Genome Detective (https://www.genomedetective.com/db/ui/login, accessed on 1 February 2023) [32] was used in default parameters to assemble the sequencing data. The resulting draft consensus sequences were subjected to BLASTn and MEGABLAST algorithms for nucleotide similarity searches in the NCBI. The alignment of the sequences was performed using the MAFFT multiple alignment program [33] version 7.388 plugin of the Geneious Prime Version 2022.2.2 (Biomatters Ltd., Auckland, New Zealand). Evolutionary history was inferred via the maximum likelihood method, using a substitution model estimated in MEGA X [34].

## 3. Results

### 3.1. Molecular Screening for Selected Viral Pathogens

Overall, 66.1% (41/62) of cats were found to be infected with at least one viral agent. Positive samples were obtained with higher frequency in animals displaying signs of enteric disease (86.2%, 25/29) than in the control group (48.4%, 16/33). Accordingly, association between the detection of viral RNA/DNA and the presence of clinical signs was fully supported by statistical analysis (*p* < 0.0027). The most frequently detected virus was FPV (35.5%, 22/62), which was found in cats with (72.4%, 21/29) and without (3.0%, 1/33) diarrhoea. FcoV was identified in 20 animals, of which 7 (24.1%) were clinically affected and 13 (39.4%) were control cats. FeChPV was detected in four diarrhoeic animals (13.8%, 4/29) and in four non-diarrhoiec (12.1%, 4/33), with an overall prevalence rate of 12.9% (8/62). Other viruses identified in cats with enteritis included FCV (13.8%, 4/29), FeKoV (3.4%, 1/29) and NoV (3.4%, 1/29). In addition, by using broadly reactive primer pairs for PyV and SaKoV, amplicons of expected size were detected in a single diarrhoeic specimen (224G/2021) (3.4%, 1/29). All cats resulted negative when screened for BoVs, FbuV DNAs and for AstVs RNA (Table 2). A statistically significant association between the occurrence of enteric disease and the presence of a viral pathogen was found for FPV (*p* < 0.00001) and FCV (*p* < 0.0426).

Out of the 29 symptomatic cats, 12 had single infection (41.3%, 12/29), 11 had dual infections (37.9%, 11/29) and 2 had triple infections (6.9%, 2/29), with the most frequent viruses involved in co-infections represented by FPV (41.3%, 12/29) and FcoV (27.5%, 8/29) (Table 3).

### 3.2. Sanger Sequencing

Direct sequencing was performed on all the amplicons obtained using broadly reactive consensus PCR protocols. In detail, on sequence analysis of the 273-bp fragment of the RdRp region (sample 232G/2021, GenBank accession no. OQ551317) amplified with NoV primer pair [26], the highest nucleotide identity (89.2–92.4%) was found to be the lion GIV NoV Pistoia-387/06/IT [35] and the GIV NoV strains previously detected in diarrhoeic cats in the US and Italy [5,15]. Sequence analysis was also carried out on amplicons obtained from the sample 224G/2021 using a pan-sakobuvirus RT-PCR protocol [29] and a PyVs degenerate PCR approach [22]. BLAST and FASTA analysis of the SaKoV positive amplicon revealed the highest identity (91.5% nt identity) to the feline Sakobuvirus, species A (SaKoV-A, strain FFUP1) [7], whilst on analysis of LIPyV DNA amplicon the highest sequence match (98.3–99.6% nt identity) was found to be in viruses recently detected in faeces of diarrhoeic cats in Canada and China [9,36].

### 3.3. ONT Sequencing

After enrichment using a SISPA protocol, eight diarrhoeic samples (168G/2020, 185G/2020, 188G/2020, 207G/2020, 212G/2020, 221G/2021, 224G/2021 and 229G/2021) were processed for high-throughput sequencing with ONT, recovering a total of 8,020,656 reads of which 1,265,516 (15.8%) had similarity to eukaryotic viruses, including parvoviruses, caliciviruses, picornaviruses, polyomaviruses, anelloviruses, papillomaviruses and morbilliviruses. A total of 41 contigs (>100 nt) were assembled and compared with viral sequences currently available on GenBank database. The longest contig was 4452 nt in length (sample 188G/2020) and showed the highest nt identity (99.6%) to a FPV strain, 3201c1/15 (KX434461), detected in 2015 in Italy in the intestinal content of a dead cat [37]. Additional 7 contigs were longer than 1000 nt, 10 contigs ranged between 400 and 1000 nt, and 23 contigs ranged between 100 and 400 nt (Table 4).

**Figure 1:**
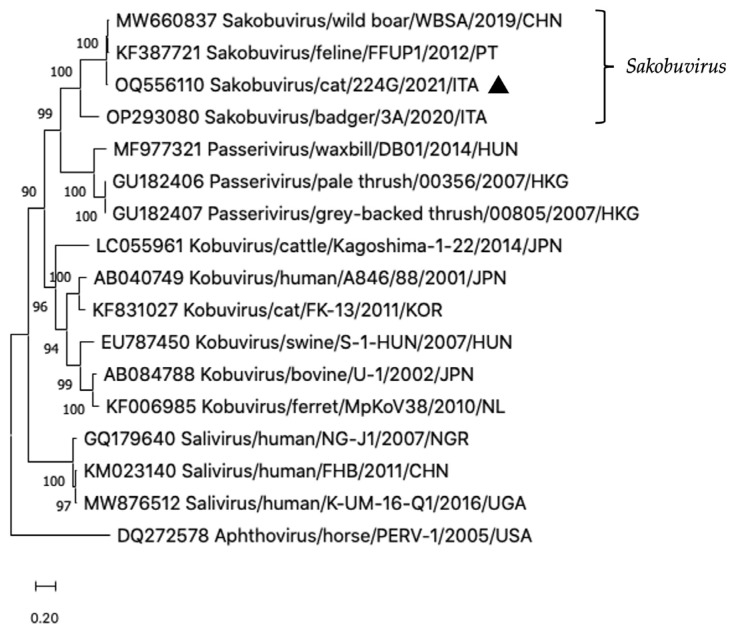
Phylogenetic analysis based on the nt sequence of the complete 2B, 2C, 3A, 3B, 3C and 3D regions of the strain SaKoV/Cat/224G/2021/ITA (OQ556110). The tree was constructed by using the maximum likelihood method based on Kimura 2-parameter model and supplying statistical support with bootstrapping of 1000 replicates. The black triangle indicates the feline strain detected in this study.

A contig of 4227 nt, sharing 90.0–90.9% nt and 96.2–96.7% amino acid [aa] identity to the feline SaKoV-A FFUP1 [7] over the 5′ partial 2A, the complete 2B, 2C, 3A, 3B, 3C, 3D genes and 3′ UTR, was detected in the sequencing library from the diarrhoeic specimen 224G/2021. On sequence analysis, the strain SaKoV/Cat/224G/2021/ITA (OQ556110) showed also high identity (88.3–89.6% nt and 94.0–95.6% aa) to another complete sakobuvirus genome (MW660837) identified in a wild boar faecal sample (data unpublished). Upon phylogenetic analysis, the Italian feline strain segregated into the genus Sakobuvirus, along with the feline and wild boar sequences, currently classified within the species Sakobuvirus A (Figure 1).

**Figure 2:**
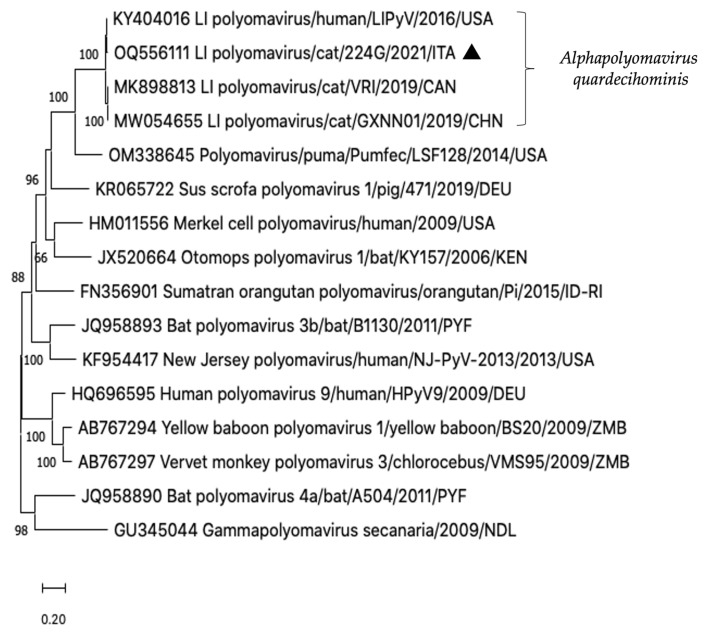
Phylogenetic tree constructed on the 5′ partial LT-Ag nt sequence of the strain LI Polyomavirus/Cat/224G/2021/ITA (OQ556111). A selection of alphapolyomaviruses of human and animal origin was included in the analysis. The evolutionary history was inferred by using the maximum likelihood method with bootstrapping (*n* = 1000) based on the Kimura 2-parameter model.

In the sample 224G/2021, an additional picornavirus contig of 152 nt was generated. This short contig shared 98.0% nt identity to the 3D gene of a novel feline hunnivirus (FeHuV), strain FeHuV-1/GZ/2017 (MF953886) identified in a cat with diarrhoea in China in 2017 [38] and proposed as a novel genotype of the species Hunnivirus A. Furthermore, in the same sequencing library, a contig of 2910 nt, comprising the 5′ partial VP1 and the 5′ partial LT-Ag encoding genes of LIPyV genome, was identified. Upon sequence analysis, the strain LIPyV/Cat/224G/2021/ITA (OQ556111) displayed the highest genetic relationship (99.4% nt identity) to the reference LIPyV sequence (NC_034253) first detected from a human skin swab in 2016 [22], whilst identities to the LIPyV genomes identified in diarrhoeic cats in Canada (MK898813) and China (MW054655) were respectively of 96.8% and 96.5% [9,36]. On phylogenetic analyses based on the partial LT-Ag nt sequence, the Italian feline LIPyV formed a single clade with the human LIPyV [22] and the two feline LIPyVs [9,36], currently classified in the species Alphapolyomavirus quardecihominis (Figure 2).

A total of 6 contigs ranging from 171 to 754 nt in length and showing the highest similarity to anelloviruses of feline origin, were found in five (168G/2020, 185G/2020, 212G/2020, 224G/2021, 229G/2021) of the eight sequencing libraries analysed, including the 224G/2021. The five contigs generated, covered fragments of the UTR and/or ORF2 gene with nt identities ranging from 73.6% to 83.7% to torque teno felis virus sequences detected in blood, serum and faecal samples from several domestic and wild felids and classified in the species Torque teno felis virus 1, 3 and 4 of the genus Etatorquevirus (family Anelloviridae) [39,40,41,42,43,44,45]. The longest contig, covering a 754 nt fragment of the ORF1 gene, showed the highest identity (70.5–71.8%) to torque teno felis viruses 4 previously identified in the faeces of pumas (puma concolor) in California (USA) and Mexico [44].

Furthermore, sequences related to felis catus papillomavirus 3 (FcaPV-3) were detected in two additional libraries (specimens no. 188G/2020 and 207G/2020). The longest contig (1654 nt) covered partially the late (L) proteins L2 and L1 coding regions of the five FcaPV-3 complete genomes available on GenBank database and to date detected in a feline bowenoid in situ carcinoma [46] in New Zeland, in feline oral squamous cell carcinoma in USA [47] and in feaces of cats in China (unpublished).The other contigs (from 310 to 560 nt in length) covered fragments of the early (E) proteins coding regions, E6, E7, E1, E2, E4 and E5. On sequence analyses, all the obtained contigs scored nt identities between 96.4% and 100% to the previously detected FcaPVs-3. A further seven short contigs of 137 to 434 nt in length related to feline morbilliviruses (FeMV) were found in a single sequencing library (cat 221G/2021). The obtained sequences encompassed regions of the N, P/V/C, F and L genes with nt identities, respectively of 82.5–96.9%, 81.6–95.8%, 78.3–97.5% and 78.1–95.6% to the FeMV genotype 1 and 2 sequences available on the database. The highest nt identities were found to FeMV genotype 1 complete genomes, identified in urine samples of cats collected in Italy [48] and Japan [49,50]. Other contigs were mapped to FPV, FCV and FeChPV sequences (Table 4), confirming the results of the molecular screening.

## 4. Discussion

In the present study, by screening cats with enteritis for a large panel of old and novel viruses, we demonstrated that the faecal virome of cats can harbour a wide diversity of viral species. A group of control cats was also included in the investigation, to evaluate the difference in detection rates between healthy and diarrhoeic animals. Overall, one or more viruses were detected in 66.1% (*n* = 41) of the 62 feline faecal samples tested with the highest prevalence rate (86.2%, 25/29) found in animals with enteric clinical signs, highlighting the role of viruses in the etiology of diarrhoea in cats. Moreover, symptomatic animals had higher co-infection rates (44.8%, 13/29), with two (37.9%, 11/29) or three viruses (6.9%, 2/29), while non-diarrhoeic cats had higher mono-infection rates (42.4%, 14/33).

Consistent with the results obtained in previous investigations [2,3,19], FPV represented the most common viral pathogen identified either alone or in co-infections with other viruses with an overall prevalence of 35.0% (22/62). The role of FPV in the etiology of feline gastroenteritis is well known [2,3,19] and was confirmed in this study, with a strong correlation between FPV infection and the presence of diarrhoea. Other viruses were found with high frequency rates, i.e., FCoV (32.2%, 20/62) and FeChPV (12.9%, 8/62) both in cats with and without diarrhoea. FCoV was detected in 24.1% (7/29) of cats with enteric disease, always in co-infection with FPV. FCoV also represented the most common enteric virus (39.4%, 13/33) in non-diarrhoeic animals. These results are in line with those obtained in previous surveys, in which FCoV was identified in cats with and without diarrhoea [51,52,53,54,55,56,57,58]. Indeed, although a role of FCoV as a pathogen contributing to enteritis has been proven on several occasions under either experimental [51,55] or field conditions [56,57,58], the infection appears also common in asymptomatic animals [52,53,54]. Interestingly, in this investigation the majority of FCoV positive cats were sampled in a narrow time span. As several studies have demonstrated that multi-cat environments facilitate the spread of the virus [59], it could be hypothesized that transmission of FCoV from infected cats to other animals occurred while they were temporally kept together in the quarantine or isolation area.

FeChPV was first identified during an outbreak of gastroenteritis in a multifacility feline shelter in Canada [8]. In a subsequent case–control study carried out in Italy, a statistically significant difference in terms of prevalence rates was observed between diarrhoeic (36.8%) and asymptomatic cats (2%) [18]. In the same investigation, FeChPV was found as a single infection only in one of the diseased animals whilst the other 13 FeChPV-positive cats were all coinfected with either FPV, or FCoV, FeKoV and NoV [18]. By converse, in a recent Australian investigation exploring the enteric virome of FPV-infected cats, FeChPV was detected only in control animals (16.7%, 6/36) [19]. In our study, FeChPV was found with similar rates in enteric diseased (13.8%, 4/29) and non-affected (12.1%, 4/33) animals. Of the four positive animals with enteritis, one was positive only for FeChPV DNA, whilst two animals were co-infected with FPV and one cat with SaKoV and LyPV.

In this study, in addition to FPV, a significant association between the occurrence of enteric disease and the presence of a viral pathogen was observed only for FCV (*p* < 0.0426). Indeed, FCV was detected only in four cats with diarrhoea, of which two animals were coinfected with FPV. Similar results were obtained in a previous investigation carried out in Italy [16], where FCV was significantly correlated with enteric signs, being found in 25.9% of stools of cats with enteritis but not in asymptomatic animals. In the 2020 study, co-infection with FCoV or with FPV, FeKoV and NoV was reportedly found in 50% of the samples [16]. Likewise, in the recent Australian study [19] FCV contigs were significantly more frequent in FPV-cases compared to healthy controls; however, they were common in mixed infections, mostly with FAstVs. Although FCV is considered primarily a feline respiratory pathogen, there are other reports in the literature on the identification of FCV in the intestinal tract of cats, either under experimental [60] or natural conditions, in diarrhoeic and healthy animals [5,13,61,62,63,64]. Considering these findings, FCV should be considered a common component of the feline enteric virome.

A role of FeNoVs as feline enteric pathogens has been suggested [5,13,14,19,64], although FeNoV sequences have also been identified by the metagenomic approach in faecal samples of clinically healthy cats [19,65]. In our survey, FeNoV genogroup IV RNA was found only in one cat with enteritis, also positive for FPV and FCoV.

Likewise, FeKoV was found only in one diarrhoeic cat, although as single viral pathogen. Although the association between infections and gastroenteritis is far from be clarified, a significant higher frequency of FeKoV detection has been reported in symptomatic cats [14,19,28,66,67]. In most cases, FeKoV was found in mixed infections with FPV, FCoV, FBoVs and FAstV [14,19,28,66,67]. In our study, one FeKoV-positive sample from a cat with enteritis was also positive for FeChPV, for SaKoV [29] and for LIPyV [22]. These findings provide the first evidence on the circulation of these two viruses in the feline population in Italy. The stools of this cat (224G/2021) were analysed by the application of a SISPA-based enrichment and nanopore sequencing, generating a large contig of SaKoV genome spanning the 3′ end (~4.2 kb). Strain SaKoV/Cat/224G/2021/ITA showed the highest identity (90.9% nt and 96.7% aa identities) to the feline SaKoV A FFUP1, the only recognized prototype member of the species Sakobuvirus A within the novel picornavirus genus Sakobuvirus. This novel picornavirus was first detected in 2012, by metagenomics analysis of faeces collected from a clinically healthy cat in Portugal [7]. In the Portuguese study, FeSaKoV was also found in co-infection with other viruses, including rotavirus, FBoV, FeAstV and picobirnavirus.

Interestingly, in sample 224G/2021 an additional viral contig was generated showing the highest identity to another picornavirus species, designated feline hunnivirus, and detected on only one occasion in cats with diarrhoea in China in 2017 [38]. In the Chinese study [38], of the three FeHuV positive samples, two were found in co-infection with either FPV or FCoV. FeHuV was proposed as a novel genotype of the species Hunnivirus A of the genus Hunnivirus, which currently includes nine genotypes identified worldwide in different animal species, including cattle, sheep and rodents [68,69,70]. The high genetic relatedness (95.1–96.6% nt and 97.0–99.2% aa) found among hunnivirus sequences detected from rodents (*Rattus norvegicus* and *Rattus tanezumi*) and cats in China [38] may suggest potential cross-species transmission [71].

The viromic approach has allowed researchers to obtain further genomic information of the LIPyV strain identified by using the PyVs degenerate PCR approach. LIPyV was first identified in cats with diarrhoea of unknown aetiology by a metagenomic analysis in Canada [9]. Three of the five cats were positive for LIPyV DNA, with one animal co-infected with FPV [9]. LIPyV sequences were subsequently identified in a faecal sample from a cat involved in another outbreak of diarrhoea and vomiting occurred in Canada, in co-infection with FBoV [8]. Subsequently, LIPyV has also been reported in China from cats with diarrhoea [8] and, more recently, from healthy cats in Australia [19]. Even more interestingly, PyVs have also been identified in human skin biopsy samples [22], leaving unanswered questions about LIPyV host range.

Feline anellovirus reads were detected in five of the eight cats analysed. These viruses have been identified, always in co-infection with other pathogens, in faecal samples of cats with or without diarrhoea [8,19,65]. The detection of anelloviruses in domestic cats dates back to 2002 in Japan, from the testing the serum of a healthy animal [39]. Epidemiological investigations testing faecal or blood samples have shown a broad circulation of anelloviruses in domestic cat populations and in a variety of wild felids [40,41,42,43,44].

Similarly, as reported in previous metagenomic investigations [8,19,72], in our study, FcaPV-3 (papillomavirus) sequence reads were found in two samples confirming the detection of these viruses in faecal samples of diarrhoeic animals, although they are recognised as a common cause of feline oral and skin neoplastic lesions [46,47,73,74].

Feline morbilliviruses have been associated with kidney disease and they can be found mostly in urine and kidney tissues of infected cats [75]. In our study feline morbillivirus reads were detected in one cat faecal sample. Likewise, in a Chinese study [76] feline morbillivirus viral RNA was detected in 4 rectal swabs of 53 stray cats which also tested positive in their urine samples. This was not completely unexpected since morbilliviruses utilize multiple pathways of infection, targeting various tissues such as the lung, kidney, gastrointestinal tract, vascular endothelium and brain [77].

Finally, despite the molecular screening using a nested RT-PCR protocol had evidenced the presence of FCoV in the sample 212G/2020, no coronavirus contigs were obtained by the viromic approach. To exclude the false positive results, the amplicon was further analysed by direct sequencing, confirming the detection of FCoV RNA (data not shown). These results may be compatible with a low-input amplification of viral genomes. Although the SISPA approach is a powerful tool for viral detection, an amplification bias which shifts the results toward the dominant genomes has been demonstrated [78]. Accordingly, further research is warranted to assess the detection limit of SISPA for low-abundance viruses.

## 5. Conclusions

Overall, in this study, along with largely known feline enteric viruses, a high number of novel viruses, still of unknown clinical relevance, were detected in faecal samples from cats with enteric signs. Large, structured epidemiological investigations are necessary to understand their pathogenic role. In addition, understanding the ecology of novel viruses of animals will be helpful to assess more precisely if and to what extent pets may pose a risk of viral infection for humans and to conceive appropriate prophylaxis plans and physical separation to limit viral transmission, especially in multi-animal environments.

## Figures and Tables

**Table 1:** List of primers used in this study.

Virus	Primer	Sequence (5′ to 3′)	Sense	Length (bp)	Tm °C	Target Gene	References
FPV	555Fw	CAGGAAGATATCCAGAAGGA	+	590	50	VP	[20]
	555ReV	GGTGCTAGTTGATATGTAATAAACA	−				
BoVs	BoVF	GCCAGCACNGGNAARACMAA	+	141	50	NS1	[4]
	BoVR	CATNAGNCAYTCYTCCCACCA	−				
FBuV	165F	CTGGTTTAATCCAGCAGACT	+	207	53	VP2	[21]
	371R	TGAAGACCA AGGTAGTAG	−				
FeChPV	FechaF1	GGTGCGACGACGGAAGATAT	+	332	58	NS1	[8]
	FechaR1	CAACACCACCATCTCCTGCT	−				
	FechaF2	GCTGCAGTTCAGGTAGCTCA	+	310			
PyVs	PyVF	CAWGCTGTRTITAGTAATA	+	240	48	LT-Ag	[22]
	PyVR	RWTTATTMACHCCITTAC	-				
FCoV	FCoVF	CTGCATGTCAAACTATTG	+	800	50	S	[24]
	FCoVR1	CTTGTGCATCAGCACTC	−				
	FCoVR2	TGCTATTATGGGACGG	−	217			
FCV	Cali1	AACCTGCGCTAACGTGCTTA	+	926	57	RdRp	[25]
	CalcR	CAGTGACAATACACCCAGAAG	−				
	CaliINT1	TGGTGATGATGAATGGGCATC	+	324	58		
	CaliINT2	ACACCAGAGCCAGAGATAGA	−				
NoVs	JV12Y	ATACCACCTATGATGCAGAYTA	+	273	37	RdRp	[26]
	JV13I	TCATCATCACCATAGAAGAG	−				
AstVs	panAstV-F1	GARTTYGATTGGRCKCGKTAYGA	+		50	RdRp	[27]
	panAstV-F2	GARTTYGATTGGRCKAGGTAYGA	+				
	panAstV-R1	GGYTTKACCCACATNCCRAA	−				
	panAstVn-F1	CGKTAYGATGGKACKATHCC	+	420	50		
	panAstVn-F2	AGGTAYGATGGKACKATHCC	+				
FeKoV	FeKoVF	CATGCTCCTCGGTGGTCTCA	+	631	55	3D^RdRp^	[28]
	FeKoVR	GTCCGGGTCCATCACAGGGT	−				
SaKoVs	SaKoV-for	GGTAGCGCGGTCGGTTGCGACCC	+	688	55	3D^RdRp^	[29]
	SaKoV-rev	CCCAGGACTGGTAGTTGTTAG	−				

**Table 2:** Viral pathogens detected in cats with or without enteric disease.

Virus	Positive Samples % (Positive/Total)	Diarrhoeic % (Positive/Total)	Non-Diarrhoeic % (Positive/Total)
FPV	35.5 (22/62)	72.4 (21/29)	3.0 (1/33)
FcoV	32.2 (20/62)	24.1 (7/29)	39.4 (13/33)
FeChPV	12.9 (8/62)	13.8 (4/29)	12.1 (4/33)
FCV	6.4 (4/62)	13.8 (4/29)	0.0 (0/33)
FeKoV	1.6 (1/62)	3.4 (1/29)	0.0 (0/33)
NoV	1.6 (1/62)	3.4 (1/29)	0.0 (0/33)
PyV	1.6 (1/62)	3.4 (1/29)	0.0 (0/33)
SaKoV	1.6 (1/62)	3.4 (1/29)	0.0 (0/33)
BoV	0.0 (0/62)	0.0 (0/29)	0.0 (0/33)
FbuV	0.0 (0/62)	0.0 (0/29)	0.0 (0/33)
AstV	0.0 (0/62)	0.0 (0/29)	0.0 (0/33)
Total positive cats	66.1 (41/62)	86.2 (25/29)	48.4 (16/33)

**Table 3:** Co-infections of viral pathogens.

Viruses	Diarrhoeic % (Positive/Total)	Non-Diarrhoeic % (Positive/Total)	Total % (Positive/Total)
FPV + FCoV	24.1 (7/29)	0.0 (0/33)	11.3 (7/62)
FPV + FCV	6.9 (2/29)	0.0 (0/33)	3.2 (2/62)
FPV + FeChPV	6.9 (2/29)	0.0 (0/33)	3.2 (2/62)
FCoV + FeChPV	0.0 (0/29)	6.1 (2/33)	3.2 (2/62)
FPV + NoV +FCoV	3.4 (1/29)	0.0 (0/33)	1.6 (1/62)
FeChPV+ PyV + SaKoV	3.4 (1/29)	0.0 (0/33)	1.6 (1/62)
Total co-infected cats	44.8 (13/29)	6.1 (2/33)	24.2 (15/62)

**Table 4:** Enteric viruses detected in the eight diarrhoeic faecal samples subjected to ONT sequencing and number of viral contigs obtained for each sample.

	Sample ID	#168G/2020	#185G/2020	#188G/2020	#207G/2020	#212G/2020	#221G/2021	#224G/2021	#229G/2021	Contigs
Viral Family	Species									
*Parvoviridae*	*Feline parvovirus*			1	2	2	1		1	7
	*Feline chaphamaparvovirus*				1		2	2		5
*Polyomaviridae*	*Lyon IARC polyomaviruses*							1		1
*Anelloviridae*	*Torque teno felis virus*	1	2			1		1	1	6
*Papillomaviridae*	*Felis catus papillomavirus*			4	4					8
*Caliciviridae*	*Feline calicivirus*	1	1	2					1	5
*Picornaviridae*	*Feline sakobuvirus*							1		1
	*Feline hunnivirus*							1		1
*Paramyxoviridae*	*Feline morbillivirus*						7			7

## Data Availability

The data supporting the findings of this study are available from the corresponding author upon reasonable request, whilst sequence data are openly available in the GenBank database.
